# Duration Selectivity in Right Parietal Cortex Reflects the Subjective Experience of Time

**DOI:** 10.1523/JNEUROSCI.0078-20.2020

**Published:** 2020-09-30

**Authors:** Masamichi J. Hayashi, Richard B. Ivry

**Affiliations:** ^1^Department of Psychology, University of California at Berkeley, Berkeley, California 94720-1650; ^2^Center for Information and Neural Networks, National Institute of Information and Communications Technology, Suita, 565-0871, Japan; ^3^Graduate School of Frontier Biosciences, Osaka University, Suita, 565-0871, Japan

**Keywords:** adaptation, duration aftereffects, fMRI, parietal cortex, time perception

## Abstract

The perception of duration in the subsecond range has been hypothesized to be mediated by the population response of duration-sensitive units, each tuned to a preferred duration. One line of support for this hypothesis comes from neuroimaging studies showing that cortical regions, such as in parietal cortex exhibit duration tuning. It remains unclear whether this representation is based on the physical duration of the sensory input or the subjective duration, a question that is important given that our perception of the passage of time is often not veridical, but rather, biased by various contextual factors. Here we used fMRI to examine the neural correlates of subjective time perception in human participants. To manipulate perceived duration while holding physical duration constant, we used an adaptation method, in which, before judging the duration of a test stimulus, the participants were exposed to a train of adapting stimuli of a fixed duration. Behaviorally, this procedure produced a pronounced negative aftereffect: A short adaptor biased participants to judge stimuli as longer and a long adaptor-biased participants to judge stimuli as shorter. Duration tuning modulation, manifest as an attenuated BOLD response to stimuli similar in duration to the adaptor, was only observed in the right supramarginal gyrus (SMG) of the parietal lobe and middle occipital gyrus, bilaterally. Across individuals, the magnitude of the behavioral aftereffect was positively correlated with the magnitude of duration tuning modulation in SMG. These results indicate that duration-tuned neural populations in right SMG reflect the subjective experience of time.

**SIGNIFICANCE STATEMENT** The subjective sense of time is a fundamental dimension of sensory experience. To investigate the neural basis of subjective time, we conducted an fMRI study, using an adaptation procedure that allowed us to manipulate perceived duration while holding physical duration constant. Regions within the occipital cortex and right parietal lobe showed duration tuning that was modulated when the test stimuli were similar in duration to the adaptor. Moreover, the magnitude of the distortion in perceived duration was correlated with the degree of duration tuning modulation in the parietal region. These results provide strong physiological evidence that the population coding of time in the right parietal cortex reflects our subjective experience of time.

## Introduction

The ability to precisely represent time is essential for optimizing perception and motor control. Various theoretical models have been proposed to account for the representation of subsecond timing, encompassing a range of mechanisms, such as functional delay lines (Ivry, 1996), neural oscillations (Treisman, 1963; Buhusi and Meck, 2005), and state-dependent neural dynamics (Buonomano and Maass, 2009). An important challenge for all of these models is to account for the fact that our perception of time is often not veridical, biased by contextual factors, such as motion (Kanai et al., 2006; Kaneko and Murakami, 2009), quantity (Dormal et al., 2006; Hayashi et al., 2013b), recent history (Pariyadath and Eagleman, 2008; Jazayeri and Shadlen, 2010; Heron et al., 2012), attention (Tse et al., 2004), and motor action (Yarrow et al., 2001; Morrone et al., 2005; Hagura et al., 2012). Although the consequences of these contextual factors have been well described behaviorally and incorporated in computational models of timing, the neural locus of such effects remains poorly understood.

Studies of perceptual adaptation have provided a powerful method to study contextual effects. Within the domain of time perception, adaptation entails repeated exposure to an adapting stimulus of a fixed duration (e.g., 250 or 750 ms), followed by the presentation of a test stimulus of variable duration (e.g., 350–650 ms) with the participants required to judge the duration of the test stimulus relative to a reference duration. This duration adaptation procedure produces a striking negative aftereffect (Heron et al., 2012): Test stimuli are more likely to be judged long after exposure to a short adaptor and judged short after exposure to a long adaptor. Moreover, the magnitude of the aftereffect is duration-specific: The aftereffect disappears for test stimuli that are quite different in duration from the adaptor. Inspired by analogous negative aftereffects observed following adaptation to perceptual features, such as orientation and motion direction (Schwartz et al., 2007), mechanistic accounts of these temporal biases have been based on the idea that the adaptor induces desensitization in duration-tuned neurons.

Although considerable study has been devoted to specifying the psychological constraints on aftereffects in duration perception (Li et al., 2015a,b; Fulcher et al., 2016; Shima et al., 2016; Maarseveen et al., 2017), the neural loci of these aftereffects has received little attention. Building on the well-established repetition suppression effect in the fMRI literature, Hayashi et al. (2015) compared the BOLD response with a visual stimulus as a function of whether a preceding stimulus had the same or different duration. Only activity in the right inferior parietal lobule, specifically the supramarginal gyrus (SMG), showed a robust repetition suppression effect, with the BOLD response lower when a specific duration was repeated. Based on the assumption that repetition suppression results from the desensitization of feature-selective cells (Grill-Spector et al., 2006; Krekelberg et al., 2006), the authors proposed that the population activity in SMG includes some form of duration tuning.

One limitation with the standard repetition suppression method, however, is that it is unclear whether the duration tuning reflects the physical or perceived duration; with a single repetition, the two are confounded. Duration adaptation procedures can allow us to determine whether a brain region is associated with physical or perceived duration because one can manipulate perceived duration while holding stimulus duration constant. In the present study, a duration adaptation procedure was tailored for the fMRI environment. We expected that the BOLD response to the test stimuli in the SMG would be context-dependent, varying as a function of the duration of the adaptor. If the duration-dependent activity in SMG is related to subjective time, the magnitude of this change would be correlated with the change in perceived duration. This result would add considerable support to the hypothesis that duration adaptation results in the desensitization of duration-tuned units, pointing to a neural correlate of the behavioral aftereffect. In contrast, if the SMG is related to physical time, we would not expect to observe a correlation between the psychophysical and physiological effects of repeated exposure to an adaptor.

## Materials and Methods

### 

#### Participants

Twenty healthy, right-handed volunteers were tested in two imaging sessions. The data from 2 of the participants were excluded from the analyses because of technical problems. Thus, the final sample was composed of 18 participants (11 males, 7 females, mean age 21.1 years, SD 3.0 years, range 18-27 years). The protocol was approved by the Institutional Review Board at the University of California Berkeley, and all participants provided informed consent.

#### Experimental design

**Figure 1.**
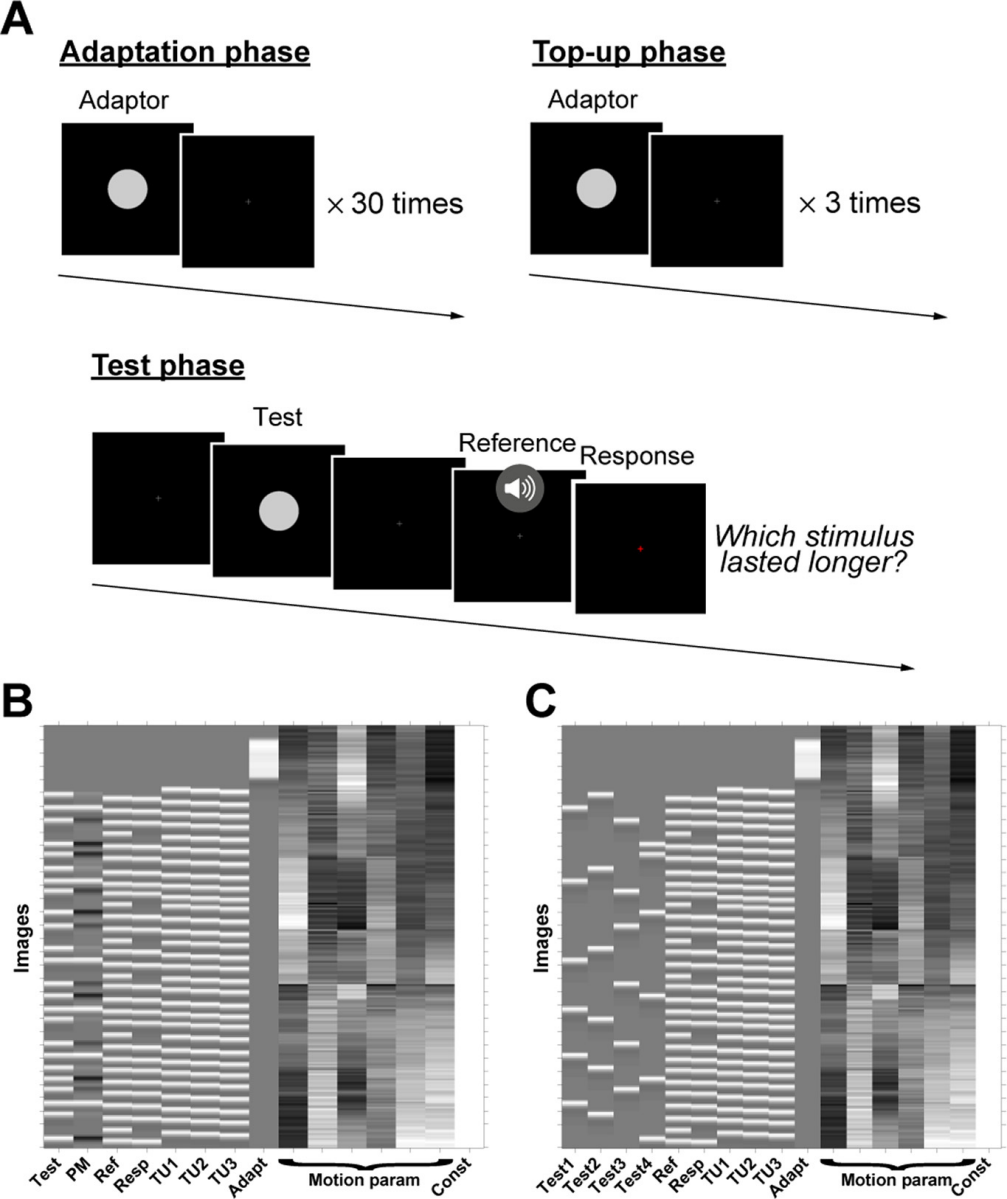
Stimulus sequence and design matrices. ***A***, Stimulus sequence. Adaptation blocks (Short and Long conditions) begin with an adaptation phase, followed by alternations between top-up and test phases. In the adaptation phase, an adaptor of a fixed duration (either 250 or 750 ms) was successively presented 30 times, with each presentation separated by a variable interval. In the top-up phase, the adaptor was presented 3 times. In the test phase, participants performed a duration discrimination task, indicating which of two stimuli was longer, a visual test stimulus of variable duration (350-650 ms) or an auditory stimulus of a fixed, reference duration (500 ms). The participant indicated their choice during a response interval, cued when the fixation cross turned red. Only the test phase was presented in the no-adaptation condition (None). Examples of design matrices for Model 1 (***B***) and Model 2 (***C***), using a single run of the long condition as an example. The regressors are listed on the *x* axes and the image number on the *y* axes (top to bottom corresponds to the first to last image, respectively). Test, Test stimuli; Ref, reference stimuli; Resp, button responses; TU1-TU3, first to third top-up stimuli; Adapt, adaptation stimuli; Motion param, head motion parameters; Const, constant term; Test 1 to Test 4, Test stimuli from shortest (Test 1) to longest (Test 4). The None (no adaptor) condition was also modeled in Model 2 in the same way as in ***C***, but the TU1, TU2, TU3, and Adapt regressors were omitted as neither adaptation nor top-up stimuli were presented.

During fMRI scanning, the participants performed a duration discrimination task, indicating which was longer: a visual stimulus of variable duration or an auditory stimulus of fixed duration (Fig. 1*A*). There were three adaptation conditions, each tested in separate scanning runs: short duration adaptation (Short), long duration adaptation (Long), and no adaptation (None). In the Short and Long blocks, each run began with an adaptation phase in which a visual stimulus (gray circle, 3.5° presented on a black background) of a fixed duration (Short = 250 ms; Long = 750 ms) was presented 30 times at the center of the display. Between presentations, the circle was replaced by a gray fixation cross (0.5° per side) for a variable duration (700, 800, or 900 ms duration, selected at random). After the 30 presentations, the fixation cross remained on the screen for a variable duration of 12.5, 13.5, or 14.5 s to signal the end of the adaptation phase.

Following the adaptation phase, the experimental program alternated between adaptation “top-up” and test phases. In the top-up phase, the gray circle adaptor was presented 3 times (duration as in the adaption phase), with the fixation cross depicted between presentations (duration 700, 800, or 900 ms). Each trial in the test phase began with the presentation of the fixation cross for a variable duration (1.5, 2.5, or 3.5 s), the test stimulus (same visual properties as adaptor but for duration of 350, 450, 550, or 650 ms), followed by the fixation cross for a variable duration (3.5, 4.5, or 5.5 s), which coterminated with an auditory stimulus (white noise, sampled at 44.1 kHz) of a fixed, 500 ms duration. Immediately after the termination of the auditory stimulus, the fixation cross turned red, initiating a 1.5 s response period during which the participant indicated, by pressing one of two buttons, which was longer: the circle (target stimulus) or auditory stimulus (reference stimulus). Responses were made with the right hand on an MRI-compatible response device (Current Designs), with the index finger used to indicate that the target stimulus was longer and the middle finger used to indicate that the reference stimulus was longer. We opted to use this cross-modal comparison task given that duration adaptation is modality-specific (Heron et al., 2012); thus, the effect of the visual adaptor should not influence the perceived duration of the auditory reference stimulus but only the perceived duration of the visual test stimulus. The instructions emphasized accuracy, with the only temporal constraint being that the response had to be entered during the 1.5 s response period.

On 20% of all trials, the test stimulus was not presented and no response was required. We included these “catch trials” to ensure that the participants paid attention to the visual test stimulus. The catch trials also allowed us to accurately estimate the evoked response to the test stimulus in the fMRI analysis by isolating the BOLD signal to this stimulus from other stimulus-evoked responses of no-interests.

The adaptation and top-up phases were not included in the no adaptation (None) blocks. Here the test phase was the same as in the Short and Long blocks, with the presentation of the circle of variable duration followed, after a variable interval, by the presentation of the auditory stimulus of fixed duration and 1.5 s response cue. To maintain the scanning duration similar to the Short and Long blocks, the duration of the fixation cross marking the start of each trial was slightly longer in the None blocks (2, 3, or 4 s).

The visual stimuli were projected by an LCD projector onto a semitransparent screen placed inside the scanner bore. The screen was viewed through a mirror mounted on the head coil. Auditory stimuli were binaurally presented through MRI-compatible S14 insert earphones (Sensimetrics). The audio output was adjusted on an individual basis to a comfortable level before starting the first imaging session, and the level was kept constant across the two sessions. Psychtoolbox (http://psychtoolbox.org) implemented in MATLAB software (MathWorks) was used to generate and present the visual and auditory stimuli. Participants were instructed to maintain fixation, either on the visual cross or gray circle at all times.

Each participant completed 12 test runs, separated into two scanning sessions with an interval of between 3 and 46 d (mean 16.3 d, SD 15.9 d) between sessions. Each session began with two None blocks, followed by four adaptation blocks. The adaptation blocks were blocked by session: Half of the participants performed four runs of the Short block during the first session and four runs of the Long block during the second session, while the other half were tested on the Short and Long blocks in the opposite order. In this manner, we collected four runs for each condition. Each adaptation block was composed of 30 trials, six for each of the test durations and six catch trials. The None blocks were composed of 45 trials, nine for each of the test durations and nine catch trials. Each fMRI run lasted 8 min 36 s.

Before entering the scanner in each imaging session, the participant performed at least one practice run composed of 20 trials of the test phase (no adaptation or top-up phases) using a laptop computer. The practice run was repeated until the participants met an accuracy criterion (at least 65% correct). All participants passed this criterion within three practice runs.

#### MRI data acquisition and preprocessing

All MRI data were acquired with a 3-Tesla Siemens Trio MRI scanner, equipped with a 12-channel head coil. For each individual, 3096 volumes of fMRI data (258 volumes × 6 runs × 2 sessions) were collected using the descending T2*-weighted gradient-echo EPI sequence with the following parameters: TR = 2000 ms, TE = 22 ms, flip angle = 50 degrees, and bandwidth = 2298 Hz/Px. The FOV was 224 × 224 mm. The digital in-plane resolution was 64 × 64 pixels, with a pixel dimension of 3.5 × 3.5 mm. To cover the entire cerebral cortex and cerebellum, 37 oblique slices were collected with 3.2 mm slice thickness and a 0.32 mm slice gap. The phase-encoding direction was along the anterior-posterior axis. High-resolution whole-brain MR images were obtained using a T1-weighted 3D MPRAGE sequence (voxel size 1.0 × 1.0 × 1.0 mm, matrix size = 256 × 256 × 256).

The first three volumes of each series of fMRI data were discarded. The remaining 255 volumes per run (a total of 3060 volumes per participant) were used in the fMRI analyses. The analyses were performed using statistical parametric mapping software (SPM12; http://www.fil.ion.ucl.ac.uk/spm/), implemented in MATLAB. Following realignment and reslicing, slice timing correction was applied to correct for variability of acquisition timing within the volume. The fMRI data were then normalized with the MNI stereotactic space using diffeomorphic anatomic registration through exponentiated lie algebra (DARTEL) algorithms in SPM12. The normalized fMRI data were subsequently smoothed in three dimensions using an 8 mm FWHM Gaussian kernel.

#### Statistical analyses

##### Behavior

For each individual, the proportions of “test stimulus longer” responses were computed for each condition and fitted by a cumulative normal function using a maximum likelihood criterion implemented on Palamedes toolbox (http://www.palamedestoolbox.org/) (Prins and Kingdom, 2009). The point of subjective equality (PSE) and slope were set as free parameters, and the other two parameters, guessing rate and lapse rate, were fixed at zero.

To compare the estimated values of PSE and slope between the three conditions (Short, Long, None), one-way repeated-measures ANOVAs (α = 0.05) were performed. When Mauchly's test indicated a violation of sphericity, the degrees of freedom were adjusted using the Greenhouse-Geisser correction. In the *post hoc* analyses, Holm-corrected *p* values were used to correct for multiple comparisons.

##### fMRI data analyses

We constructed two GLMs for analyzing the fMRI data for each individual. The first model (Model 1; Fig. 1*B*) was aimed at identifying brain areas that showed a duration-selective attenuation in the BOLD response for test stimuli following duration adaptation. Only the data from the adaptation conditions (S and L conditions, only) were included in this analysis. The second model (Model 2; Fig. 1*C*) was designed to extract the BOLD responses for each test duration from the clusters identified in Model 1. To compare the BOLD activities for each duration and condition, Model 2 included the data from the Short, Long, and None conditions.

The offsets of test stimuli, onsets of the reference stimuli, button responses, onsets of top-up stimuli, and onsets of adaptation stimuli in the adaptation phase were included in Model 1. We opted to model the offset responses of the test stimuli rather than their onsets because duration information is most salient at the end of each test stimulus (Hayashi et al., 2015).

To analyze the attenuation in the BOLD response for the test stimuli, we added a parametric modulation (PM) term for the test stimuli regressors. The modulation parameter was determined by a deviation ratio, computed by taking the difference between the longer and shorter duration stimuli, and dividing by the shorter duration stimulus (Hayashi et al., 2015). Specifically, in the Short condition, the shorter duration was the adaptor duration (250 ms) and the longer duration was the test duration (350, 450, 550, or 650 ms); in the Long condition, the shorter duration was the test duration (350, 450, 550, or 650 ms) and the longer duration was the adaptor duration (750 ms). Thus, the modulation parameters were 0.4, 0.8, 1.2, and 1.6 for the four test durations in the Short condition, and 1.14, 0.67, 0.36, and 0.15 for the four test durations in the Long condition. These values were mean-adjusted to zero, and then entered as the PM parameter for the Short and Long conditions. We expected that the modulation term would capture the modulation of duration tuning, assuming the BOLD response is attenuated when the difference in duration between the adaptor and test duration is small, and gradually become smaller when the difference in duration becomes larger.

The onsets of the three presentations of the top-up stimulus were modeled by separate regressors in Model 1. Motion parameters estimated in the realignment procedure were also included in Model 1 to regress the potential motion-induced signal fluctuations. In summary, seven independent regressors with one PM term and 6 regressors of no-interest (the motion parameters) were included in Model 1 for the Short and Long conditions.

Model 2 was similar to Model 1, except that the four test durations were modeled by separate regressors instead of the PM terms. By separating the regressors for the test durations, this model allows us to obtain estimates of the BOLD response for each test duration separately. The fMRI data from the None condition were modeled in the same way as for the Short and Long conditions, but without regressors for the top-up and adaptation phases. For all three conditions, motion parameters, estimated in the realignment procedure, were again included to regress out motion-induced signal fluctuations. Thus, Model 2 included 10 independent regressors and 6 regressors of no-interest (the motion parameters) for the Short and Long conditions and 6 independent regressors and 6 regressors of no-interest for the None condition.

Event durations of all regressors of interests were set to zero and convolved by a canonical HRF. The models were high-pass filtered (128 s), and a constant term was included to capture baseline effects.

Our *a priori* hypothesis was that duration adaptation would result in repetition suppression in the right SMG (Hayashi et al., 2015); as such, our primary analysis focused on this region. To perform an ROI analysis in the right SMG, we created an anatomically defined mask of the right SMG using WFU PickAtlas software (https://www.nitrc.org/projects/wfu_pickatlas/).

To make population inferences for the effect of duration adaptation on the test stimuli, we performed a group-level analysis with a random effects model. We constructed a full-factorial model with the individuals' contrast images (i.e., the parameter estimates) using the PM terms for the Short and Long conditions computed by Model 1. In the statistical analysis, we applied the ROI mask to restrict the search volume within the right SMG. A statistical threshold of *p* < 0.05, familywise error corrected at the cluster level (defined by *p* < 0.001 uncorrected at the voxel level), was used as the criterion for statistical significance. To further explore brain areas that showed an effect of duration adaptation, we also performed the same analysis without the mask. A slightly liberal threshold of *p* < 0.001 uncorrected at voxel level (cluster size *k* > 30 voxels) was used as the criterion for statistical significance.

Parameter estimates were extracted from the statistically significant clusters and averaged across the voxels. The voxel-by-voxel parameter estimates for the PM terms were obtained from Model 1, and the parameters for each stimulus duration were obtained from Model 2, estimated in the individual-level analyses. To assess the changes in the parameter estimates across test durations and the three conditions (Short, Long, None), we performed a two-way repeated-measures ANOVA using within-factors of Condition and Test Duration (α = 0.05).

##### Correlation analyses

Two types of correlation analyses were performed. The first involved correlations between the magnitude of the behavioral aftereffect and the degree of the attenuation in the BOLD response (i.e., BOLD aftereffect size); the second involved correlations of the BOLD aftereffect between different pairs of brain regions. The differences in the PSE estimates between the Short and Long conditions were operationalized to indicate the magnitude of the behavioral aftereffect. The magnitude of the BOLD aftereffect was operationalized as the sum of the regression coefficients for the PM terms in Model 1. Both types of correlation analyses were statistically evaluated by computing the Spearman's correlation (α = 0.05, two-tailed). We examined the robustness of the correlations by computing 95% CIs, based on a bootstrapping method (10,000 samples) using the Robust Correlation Toolbox (Pernet et al., 2012).

## Results

### Behavioral negative aftereffects

**Figure 2.**
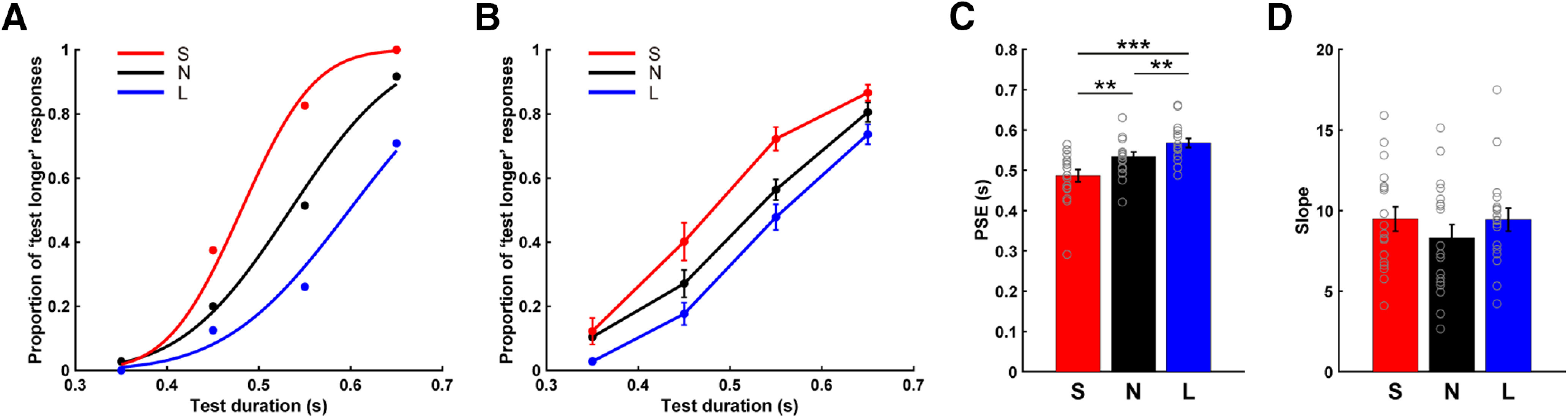
Behavioral results. Task performance of a representative participant (***A***) and the average performance for all participants (***B***). Data show the percentage of trials for the three conditions in which the test stimulus was judged to be longer than the reference stimulus. Estimates of the PSE (***C***) and slope (***D***) from the psychometric fitting procedure. Red represents Short condition (S); black represents None condition (N); blue represents Long condition (L). Gray circles on the bar graphs represent individual data. Error bars indicate SEM. **p < 0.01, ***p < 0.001.

The behavioral data showed that participants exhibited a systematic increase in the proportion of “test longer” responses as the test duration increased, indicating that participants were attending to the task (Fig. 2*A*,*B*). To quantify the effect of perceptual adaptation on perceived duration, we fit the individual response functions with a psychometric function to estimate the PSE, a measure of bias, and slope, a measure of variability (Fig. 2*C*,*D*). A one-way repeated measures ANOVA revealed that the PSE estimates differed between the three conditions (*F*_(2,34)_ = 20.300, *p* < 0.001, η^2^ = 0.544). *Post hoc* comparisons confirmed that, relative to the None condition (mean ± SD; 533 ± 50 ms), the PSE was lower in the Short condition (487 ± 62 ms; *t* = 3.490, *p* = 0.006, Cohen's *d* = 0.822) and higher in the Long condition (568 ± 46 ms; *t* = −3.160, *p* = 0.006, Cohen's *d* = −0.745). Thus, the duration of the test stimuli was overestimated following adaptation to the 250 ms adaptor and underestimated following adaptation to the 750 ms adaptor, the signature of a negative aftereffect.

**Figure 3.**
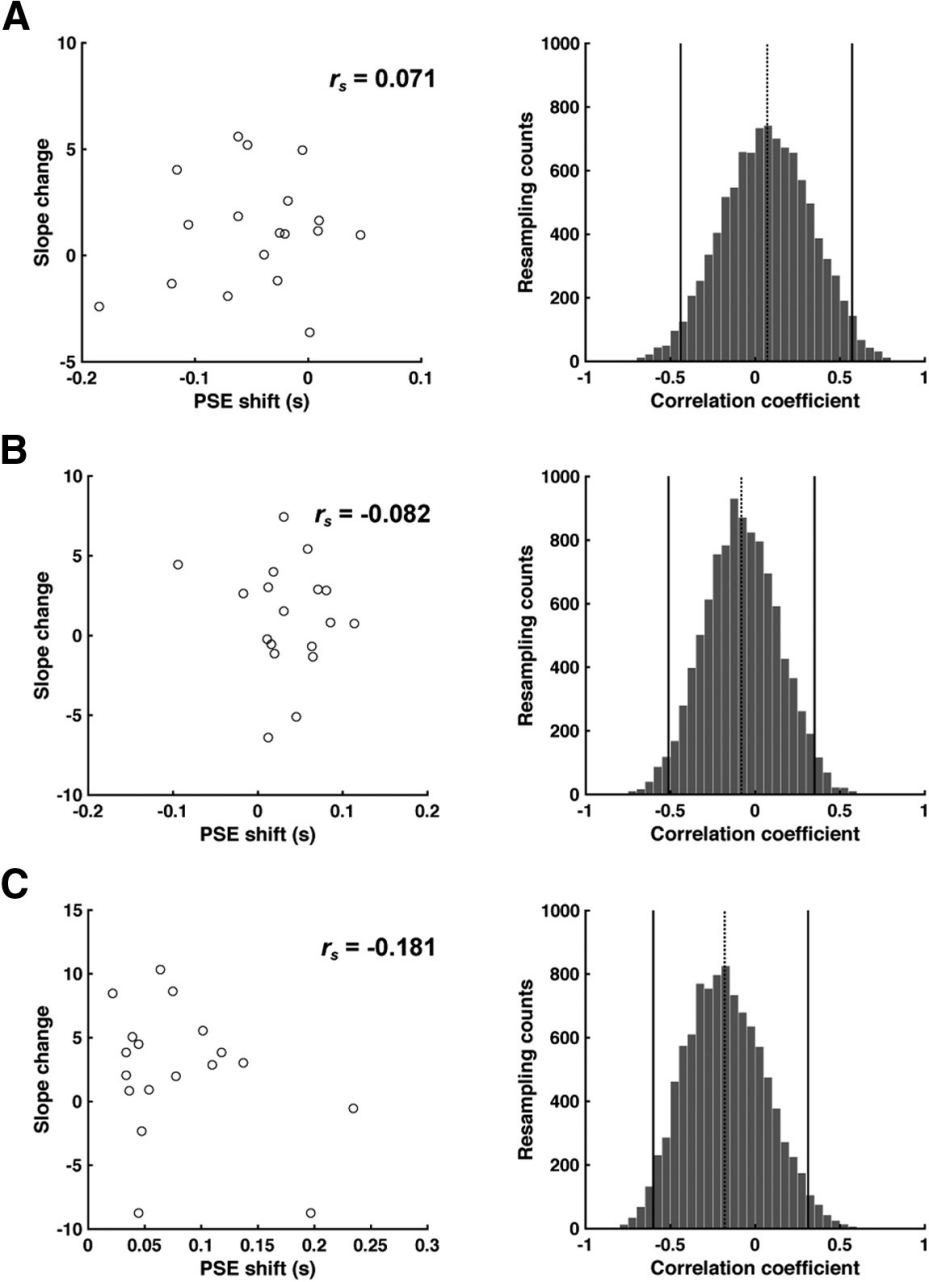
The effect of adaptation on bias and variability is not correlated. Correlations between the shift in PSE and change in slope between the Short and None conditions (***A***), Long and None conditions (***B***), and Long and Short conditions (***C***). The *r*_s_ values in each panel indicate Spearman's correlation. Right column represents distribution of correlation coefficients estimated by bootstrap method. Solid lines indicate 95% CI. Dotted line indicates Spearman's correlation from the corresponding panel in the left column.

The slopes of the psychometric functions were not different between conditions (*F*_(2,34)_ = 1.554, *p* = 0.226, η^2^ = 0.084; Short: 9.479 ± 3.109; Long: 9.439 ± 2.941; None: 8.311 ± 3.405) (*F*_(2,34)_ = 1.554, *p* = 0.226, η^2^ = 0.084). Moreover, the magnitude of the changes in the PSE and slope values were not correlated across individuals (Short and None: *r*_s_ = 0.071, *p* = 0.789; Long and None: *r*_s_ = −0.082, *p* = 0.717; Long and Short: *r*_s_ = −0.181, *p* = 0.475) (Fig. 3, left column). To examine the robustness of this analysis, we computed 95% CIs for the distribution of correlation coefficients, taking 10,000 samples in a bootstrapping method. This analysis confirmed the lack of correlation between the two psychophysical measures given that the distributions included zero (Short and None: 95% CI = −0.440–0.571; Long and None: 95% CI = −0.512–0.349; Long and Short: 95% CI = −0.601–0.311; Fig. 3, right column). Thus, the size of the negative aftereffect was independent of the participants' variability in making the psychophysical judgments.

### Neural adaptation and neurobehavioral correlation in the right SMG

We hypothesized that adaptation would produce an attenuation in the BOLD response, and in particular, that this effect would be most evident for the test stimuli that are similar in duration to the adapting stimulus duration. Motivated by prior studies on the cortical representation of duration (Wiener et al., 2012; Hayashi et al., 2015), our *a priori* prediction was that the BOLD attenuation would be evident in right SMG.

**Figure 4.**
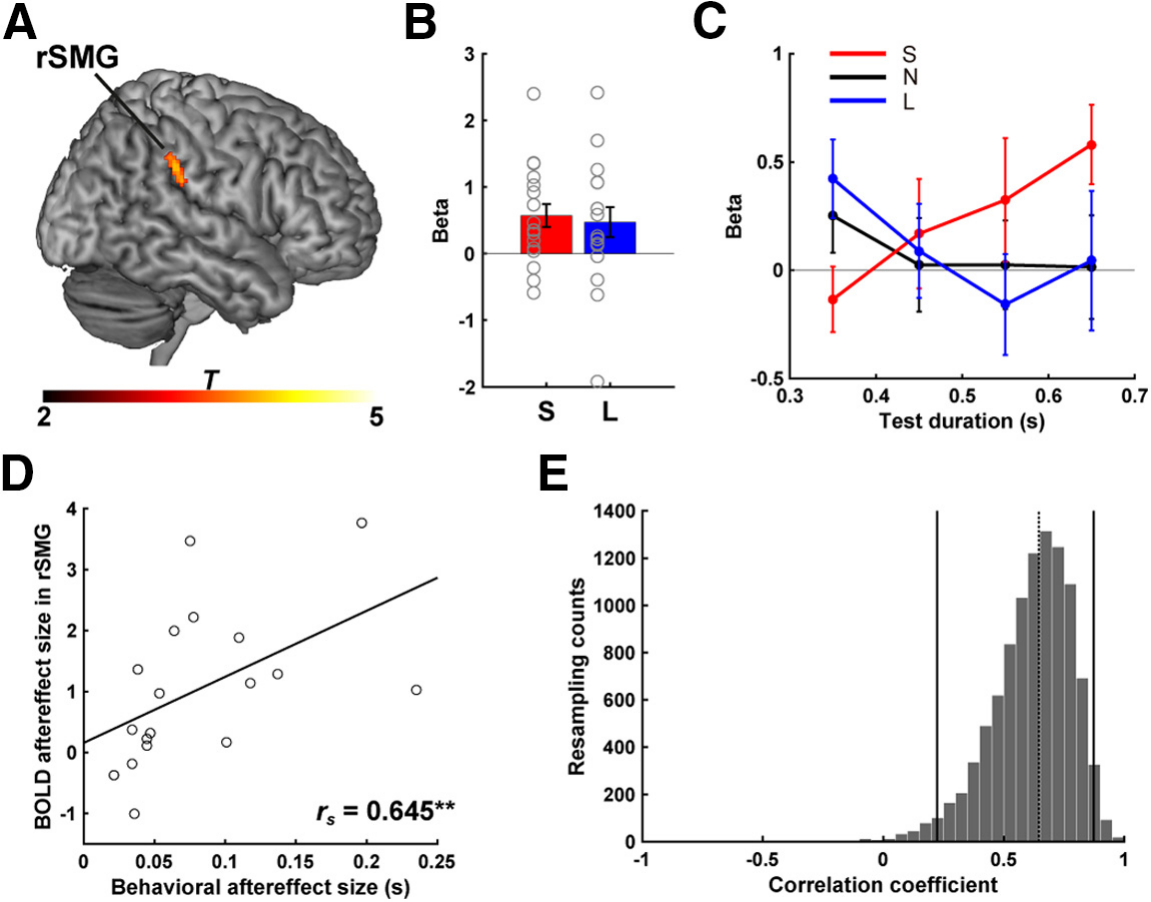
ROI analysis of SMG. ***A***, Cluster in right SMG that exhibited a BOLD aftereffect, with the BOLD response to the test stimuli differentially modulated during the adaptation runs (Short and Long conditions). Color scale represents *t* values. Mean regression coefficients of the PM term in the GLM (Model 1) in the adaptation runs (***B***) and for each test duration, extracted from Model 2 (***C***). Colors, gray circles, and error bars are the same as in Figure 2. ***D***, Correlation between behavioral aftereffect and modulation of BOLD response as a function of test stimulus duration in right SMG. Least-square fit line is plotted. ***E***, Distribution of correlation coefficients estimated by bootstrap method. Solid lines indicate 95% CI. Dotted line indicates Spearman's correlation for the neurobehavioral effect shown in ***D***.

Consistent with this prediction, the ROI analysis (Model 1) revealed modulation of duration tuning following adaptation in a cluster of voxels in right SMG, time-locked to the offset of the test stimuli (*p* < 0.05 familywise error cluster-level corrected, defined by *p* < 0.001 uncorrected at voxel level) (Fig. 4*A*; Table 1). The regression coefficients (β values in Fig. 4*B*), reflective of the PM term in the GLM (see Materials and Methods, Model 1), indicate that the degree of modulation was dependent on the similarity between the test stimulus duration and adaptor duration. That is, for the Short condition, the BOLD response was more attenuated for relatively shorter test stimuli; and for the Long condition, the BOLD response was more attenuated for relatively longer test stimuli. This adaptor-dependent modulation can be seen in Figure 4*C*, where the β values for each test stimulus are displayed (Model 2).

**Table 1.** Parameters of clusters exhibiting duration adaptation in the ROI analysis of right SMG and the whole-brain analysis^a^

Cluster size	Location	Side	MNI coordinates			*Z*
			*x*	*y*	*z*	
49	SMG	R	66	−34	34	3.58^b^
49	MOG	L	−42	−80	24	3.90
38	MOG	R	50	−70	26	3.58

*^a^*One cluster that appeared in the white matter was omitted from this table.

*^b^*SMG cluster found in the ROI analysis.

To statistically evaluate these effects, we analyzed the β values for each test stimulus with a two-way repeated-measures ANOVA, using the factors Condition and Test Duration. The results showed a significant interaction (*F*_(6,102)_ = 2.747, *p* = 0.016, η^2^ = 0.139), with no main effects of Condition (*F*_(2,34)_ = 0.282, *p* = 0.756, η^2^ = 0.016) or Test Duration (*F*_(3,51)_ = 0.652, *p* = 0.586, η^2^ = 0.037). The BOLD response varied as a function of the test duration (simple main effects of test duration) in the Short condition (*F*_(3,153)_ = 3.370, *p* = 0.021), and a similar trend was observed in the Long condition (*F*_(3,153)_ = 2.182, *p* = 0.095). In contrast, the β values were similar for all four test durations in the None condition (*F*_(3,153)_ = 0.501, *p* = 0.682). Together, these results provide support for the hypothesis that neural activity in the right SMG is representative of duration-tuned neural populations.

Having observed negative aftereffects in the participants' behavior and duration tuning modulation in right SMG, we next examined the relationship between these measures. We used the difference between the PSE values from the Short and Long conditions to quantify the negative behavioral aftereffect; to quantify the duration tuning modulation (i.e., BOLD aftereffect), we took the degree of modulation of the BOLD response in right SMG across the four test stimuli (sum of β values shown in Fig. 4*B*). Importantly, we found a strong correlation between the behavioral and physiological measures (*r*_s_ = 0.645, *p* = 0.004; Fig. 4*D*). To examine the robustness of this correlation, we computed 95% CIs for the distribution of correlation coefficients by taking 10,000 samples in a bootstrapping method. This analysis showed that the observed correlation coefficient was reliably different from zero (95% CI = 0.223–0.873, *p* = 0.006; Fig. 4*E*). These results are consistent with the hypothesis that the modulation of subjective time following duration adaptation is related to the degree of modulation by the adaptor of the BOLD response in right SMG to the test stimuli.

### Neurobehavioral correlations in the bilateral MOG

**Figure 5.**
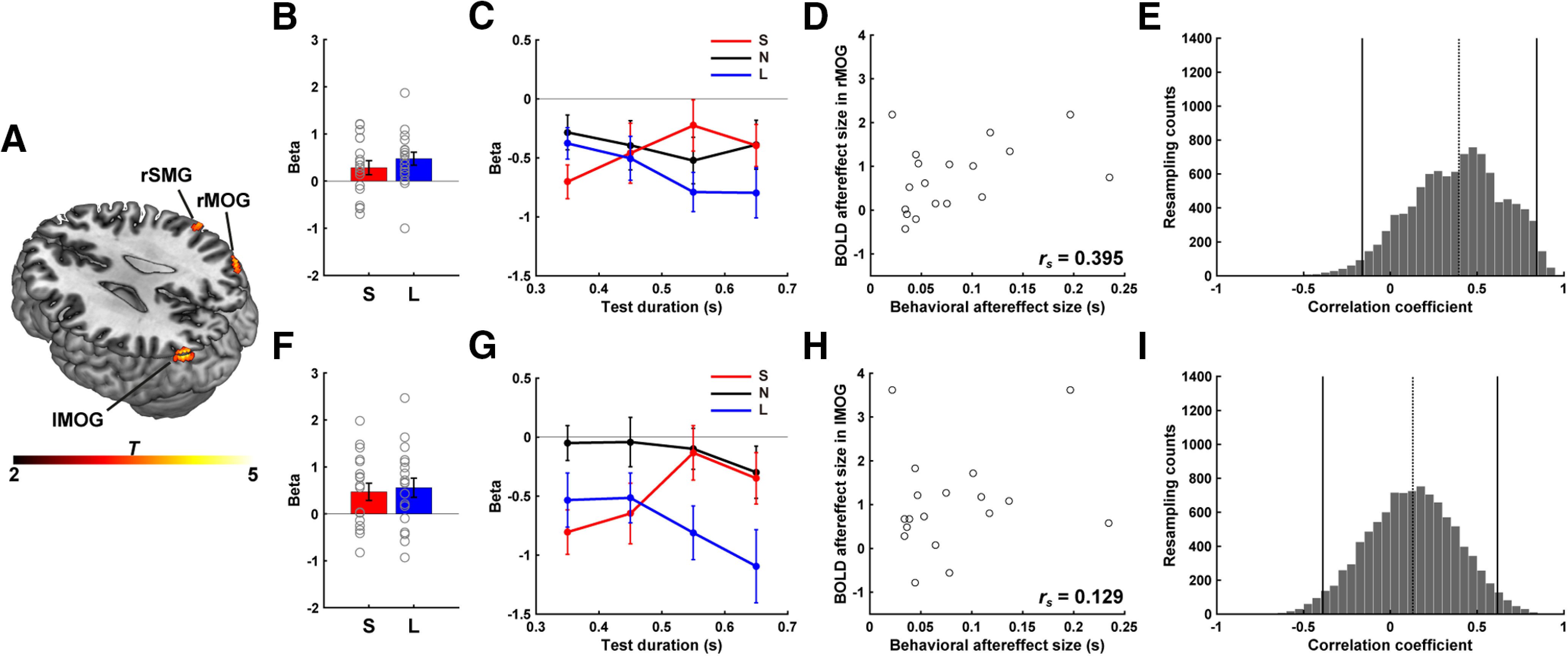
Whole-brain analysis of neural adaptation. ***A***, In addition to right SMG, clusters within MOG in the right and left hemispheres exhibited a BOLD aftereffect in response to the test stimuli during the adaptation runs. Color scale represents *t* values. Detailed analyses of the BOLD response in right MOG (***B***–***E***) and left MOG (***F***–***I***). Mean regression coefficients of the PM term in the GLM (Model 1) in the adaptation runs (***B***,***F***) and for each test duration, extracted from Model 2 (***C***,***G***). Colors, gray circles, and error bars are the same as in Figure 2. ***D***, ***H***, Neurobehavioral correlations in the right and left MOG, depicted as in Figure 4*D*. ***E***, ***I***, Distribution of correlation coefficients estimated by bootstrap method. Solid lines indicate 95% CI. Dotted line indicates Spearman's correlation for the neurobehavioral effect (***D***,***H***).

We also performed a whole-brain analysis to identify other cortical and subcortical regions that exhibit duration tuning modulation following adaptation to a stimulus of a fixed duration (Model 1). Using a liberal threshold (*p* < 0.001, uncorrected at voxel level), this analysis identified only three clusters: one in the right SMG area described previously, and the other two in middle occipital gyrus (MOG), bilaterally (Fig. 5*A*; Table 1). The main effect of Condition was significant in the left MOG (*F*_(2,34)_ = 3.879, *p* = 0.030, η^2^ = 0.186) but not in the right MOG (*F*_(2,34)_ = 0.817, *p* = 0.450, η^2^ = 0.046), whereas the main effect of Test Duration was not significant in either region (Right MOG: *F*_(3,51)_ = 0.350, *p* = 0.789, η^2^ = 0.020; Left MOG: *F*_(3,51)_ = 1.873, *p* = 0.146, η^2^ = 0.099). Most important, as with SMG, the interaction term was significant for both clusters (Right MOG: *F*_(6,102)_ = 2.478, *p* = 0.028, η^2^ = 0.127; Left MOG: *F*_(6,102)_ = 3.455, *p* = 0.004, η^2^ = 0.169) (Fig. 5*B*,*C* for the right MOG, and Fig. 5*F*,*G* for the left MOG). Although the β values in the right and left MOG (Fig. 5*C*,*G*) were negative, the sign is not important given the arbitrary baseline used in the event-related fMRI design.

The main effect of Test Duration was significant in the left MOG in both the Short and Long conditions (Short: *F*_(3,153)_ = 4.183, *p* = 0.008; Long: *F*_(3,153)_ = 3.447, *p* = 0.019) and approached significance in right MOG for both conditions (Short: *F*_(3,153)_ = 2.196, *p* = 0.093; Long: *F*_(3,153)_ = 2.474, *p* = 0.066). As with SMG, there was no effect of Test Duration in the None condition (right MOG: *F*_(3,153)_ = 0.538, *p* = 0.658; left MOG: *F*_(3,153)_ = 0.665, *p* = 0.575). In combination with the GLM analyses, these results indicate that the degree of neural adaptation in left and right MOG was dependent on the duration of the adaptor, with the effect greatest for test stimuli most similar in duration to the adaptor.

We performed the neurobehavioral correlation for right and left MOG. In contrast to SMG, the magnitude of the behavioral aftereffect was not correlated with the magnitude of the BOLD aftereffect in either MOG cluster (right MOG: *r*_s_ = 0.395, *p* = 0.104, Fig. 5*D*; left MOG: *r*_s_ = 0.129, *p* = 0.610, Fig. 5*H*). The bootstrap analyses confirmed that the distribution of correlation coefficients included zero (right MOG: 95% CI = −0.162 to 0.843, *p* = 0.175, Fig. 5*E*; left MOG: 95% CI = −0.389 to 0.617, *p* = 0.629, Fig. 5*I*). Thus, although we observed a BOLD aftereffect in MOG, the magnitude of the response in left and right MOG was not correlated with the changes in perceived duration following adaptation.

We recognize that this last point is based on a null result: The neurobehavioral correlations for right and left MOGs may be qualitatively similar to that observed in right SMG, even if not statistically significant. To address this question, we used a bootstrap procedure to compare the neurobehavioral correlation coefficients of right SMG, right MOG, and left MOG. We used this analysis to estimate the distribution of the difference in correlation coefficients between each brain region pair, taking 10,000 samples. The estimated distributions were evaluated by assessing whether the 95% CI included zero. This analysis indicated that the neurobehavioral correlations were similar across the three regions, with each distribution including zero (right SMG vs right MOG: 95% CI = −0.393 to 0.914, *p* = 0.512; right SMG vs left MOG: 95% CI = −0.116 to 1.115, *p* = 0.123; right MOG vs left MOG: 95% CI = −0.017 to 0.556, *p* = 0.062). Thus, while the changes in perceived durations were strongly associated with modulation of the BOLD response in right SMG, a similar pattern is also observed in the two occipital regions.

### Correlations of BOLD aftereffect size between brain regions

**Figure 6.**
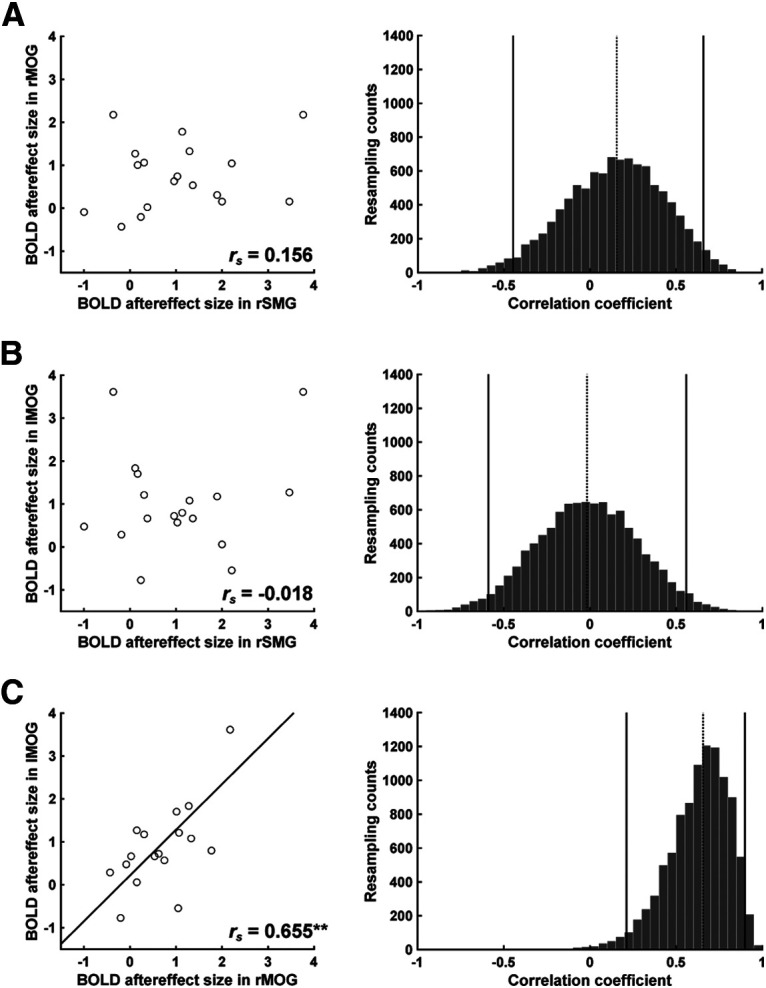
Comparison of the BOLD aftereffect across brain regions. Left column represents the correlations for each pairwise comparison of the three areas exhibiting a BOLD aftereffect as a function of the duration of the test stimulus: ***A***, Right SMG and right MOG. ***B***, Right SMG and left MOG. ***C***, Right MOG and left MOG. The *r*_s_ values indicate Spearman's correlation. Solid lines indicate the least-square fit lines. Right column represents distribution of correlation coefficients estimated by bootstrap method. Solid lines indicate 95% CI. Dotted line indicates Spearman's correlation for the neurobehavioral effect shown in corresponding left column panel.

In the final analysis of the BOLD aftereffects, we examined the correlations between the size of this effect in right SMG, right MOG, and left MOG. Positive correlations would suggest that the effect in one area might be driven by the effect in a different area. The magnitude of the BOLD aftereffect in right and left MOG was correlated (*r*_s_ = 0.655, *p* = 0.003), a result confirmed with the bootstrap method (95% CI = 0.212-0.898, *p* = 0.008) (Fig. 6*C*). Interestingly, the BOLD aftereffect in both of these areas was not correlated with that observed in right SMG (right SMG–right MOG: *r*_s_ = 0.156, *p* = 0.537; right SMG–left MOG: *r*_s_ = −0.018, *p* = 0.945). This null result was confirmed with the bootstrap method (right SMG–right MOG: 95% CI = −0.447–0.660, *p* = 0.610; right SMG–left MOG: 95% CI = −0.589–0.558, *p* = 0.964) (Fig. 6*A*,*B*).

Based on the overall pattern of results here, we speculate that the BOLD response in right and left MOG may reflect similar input from early visual cortex (and interhemispheric cross-talk) with these areas responsive to physical duration, with a more modest effect of perceived duration. The duration tuning modulation in SMG appears to be independent of the modulatory effects in MOG, suggesting that this region might be a point of convergence of different inputs that underlie our subjective experience of time.

## Discussion

To examine the neural mechanisms underlying subjective time perception, we used a duration adaptation procedure that allowed us to distinguish between the subjective time of a visual event and its physical time. The neuroimaging results showed that the adaptation procedure produced duration-selective attenuation of the BOLD response in right SMG, and that the degree of attenuation was correlated with the size of the behavioral aftereffect. These results suggest that activity in the right SMG reflects our subjective experience of time.

A prominent model of an fMRI adaptation, the fatigue model, proposes that the decrease in the BOLD response following adaptation to a specific stimulus feature reflects reduced neural activity because of the repetitive activation of neural populations that are tuned to the repeated stimulus feature (Grill-Spector et al., 2006; Alink et al., 2018). Applying this logic used to interpret adaptation effects in the spatial domains, the attenuation of the BOLD response (i.e., duration tuning modulation) observed in the current study can be interpreted as providing evidence for the existence of duration-tuned neural populations in the human brain (Heron et al., 2012). Namely, following repeated exposure to a stimulus of a fixed duration, populations tuned to that duration become fatigued, producing a reduction in the BOLD response.

Importantly, we observed a correlation between our physiological measure of neural adaptation and our behavioral measure of subjective time: Participants who showed the strongest modulation of duration tuning in right SMG also showed the largest behavioral aftereffect. Using other visual properties, adaptation methods have shown similar neurobehavioral correlations for motion perception in MT+ (Huk et al., 2001), biological motion in pSTS (Thurman et al., 2016), and facial expression and identity in anterior medial temporal cortex (Furl et al., 2007; Cziraki et al., 2010). These correlations have been taken to provide strong, albeit indirect, evidence of a causal role of the neural area with its associated behavior. Here we propose that the correlation between the behavioral aftereffect and the degree of duration tuning modulation in SMG is compatible with the hypothesis that the bias in perceived duration following duration adaptation arises from altered response properties of duration-tuned neural populations.

A prominent computational model of duration adaptation is based on the idea that aftereffects result from a gradient of desensitization across a bank of neurons tuned to different durations, with the center of desensitization at the duration of the adaptor (Heron et al., 2012). The resulting variation in sensitivity leads to a shift in the population response away from the adapted duration, which results in a negative aftereffect (Schwartz et al., 2007). By showing the correlation between the effects of the adaptor on behavior and the BOLD response, our study provides the first physiological evidence in support of this model, with the results pointing to the right SMG as the locus of duration-tuned neural populations. Our results are also consistent with other models of fMRI adaptation, such as the idea that adaptation results in the sharpening of tuning curves (Grill-Spector et al., 2006). Electrophysiological recordings may be essential for evaluating these different neural models of fMRI adaptation in the time domain.

The neurobehavioral correlation is also relevant to the current debate concerning where our subjective experience of time arises from within the temporal processing hierarchy (Shima et al., 2016; Li et al., 2017a; Heron et al., 2019). Some researchers have proposed that duration channels are located in early processing stages given psychophysical evidence showing that duration aftereffects exhibit modality (Heron et al., 2012; Li et al., 2019) and, with visual stimuli, some degree of spatial specificity (Fulcher et al., 2016; see also Johnston et al., 2006). In contrast, a later-stage hypothesis is supported by studies showing that the aftereffects in the perceived duration of visual stimuli lack orientation (Li et al., 2015b) and position specificity (Li et al., 2015a; see also Burr et al., 2007; Anobile et al., 2019). The right SMG locus observed in the present study would be more consistent with the later-stage account. It may be that duration channels in this area constitute a read-out mechanism that integrates temporal information arising from neural activity in early sensory areas. Interestingly, temporoparietal junction, which includes SMG, has been associated with our awareness of visuospatial information (Beauchamp et al., 2012). Although highly speculative, our findings may point to a more general role of temporoparietal junction in awareness, one associated with our subjective experience of not only spatial, but also temporal, information.

In our previous study on duration perception, we had observed a suppression of the BOLD response in right SMG when a visual stimulus was repeated for the same duration (Hayashi et al., 2015). In both our ROI and exploratory whole-brain analysis, the present results replicate and extend this finding. The initial study did not allow us to differentiate between subjective and physical time because it involved paired stimuli (i.e., a reference stimulus and a test stimulus, without any adaptation phase), a procedure that does not produce a distortion of perceived durations (Heron et al., 2012; Li et al., 2017b). Moreover, the two stimuli were presented in the same modality; and thus, any distortion would impact both stimuli. By using an established duration adaptation procedure (Heron et al., 2012; Li et al., 2015a,b; Fulcher et al., 2016; Maarseveen et al., 2017), we were able to measure duration aftereffects in the MRI environment, observing the correlation between duration-sensitive activity in right SMG and the subjective experience of time.

The right lateralized effect observed here in the parietal cortex reported is consistent with our previous study of duration-selective repetition suppression (Hayashi et al., 2015). Lesion studies, either involving neurologic patients (Harrington et al., 1998) or transient disruption from TMS (Bueti et al., 2008; Wiener et al., 2010a), also have pointed to a greater involvement of the right parietal lobe relative to the left in duration perception. On the other hand, left parietal cortex has been implicated in temporal prediction tasks in which temporal information can facilitate perception and action (Wiener et al., 2010c). It is possible that laterality patterns are related to the distinction between explicit and implicit timing (Coull et al., 2013; Breska and Ivry, 2016), where the former refers to tasks where the task goal focuses on the temporal property of the stimulus while the latter refers to tasks in which the task goal focuses on nontemporal properties (e.g., detection, stimulus identification). It will be interesting to develop adaptation methods for implicit timing tasks to test this hypothesis.

In addition to right SMG, we also observed a BOLD aftereffect in left and right MOG following duration adaptation, a result that would suggest that MOG also contains duration-tuned neural populations. The relationship between the physiological changes in MOG and the behavioral aftereffect is problematic: The neurobehavioral correlations were not significant for either area, but they were in the same direction as that observed in SMG. Moreover, the neurobehavioral correlation in SMG was not significantly stronger than that observed with either left or right MOG. Although we can only speculate, one possible interpretation might be that activity in bilateral MOG is less sensitive to the history of temporal information.

Interestingly, the degree of the BOLD aftereffect was correlated between right and left MOG (Fig. 6*C*), consistent with the foveal presentation of the stimuli, but the BOLD aftereffect in neither area correlated with the degree of BOLD aftereffect in right SMG. Whether the SMG and MOG are functionally related is still an open question. Future studies applying a functional connectivity analysis to a suitable neuroimaging experiment may provide insight into the issue of whether the SMG and MOG interact with each other.

Previous studies have pointed to a broad network of neural regions engaged in temporal processing, including the supplementary motor area, inferior frontal gyrus, cerebellum, and basal ganglia (Wiener et al., 2010b; Hayashi et al., 2014, 2018; Protopapa et al., 2019; Harvey et al., 2020). Although the ROI approach used here was designed to focus on right SMG, it is noteworthy that none of these cortical or subcortical areas showed duration-selective attenuation of the BOLD response in the exploratory, whole-brain analysis. These areas may contribute to other aspects of performance on timing tasks; alternatively, they may use different coding mechanisms for timing that are insensitive to our adaptation manipulation. For example, activity in supplementary motor area exhibits ramping neural activity (van Rijn et al., 2011), and inferior frontal region has been shown to represent time in a categorical manner (e.g., longer or shorter) (Hayashi et al., 2013a), both signatures that may be more indicative of decision- and response-relevant representations. One important direction for future research would be to dissociate different processing operations required for making temporal judgments.

In conclusion, the present study demonstrates that physiological activity in right SMG associated with temporal processing is contextually sensitive, with exposure to an adapting stimulus-modulating duration tuning in this area. Moreover, the degree of the BOLD aftereffect was predictive of behavioral performance, with individuals who showed the largest tuning modulation also exhibiting the largest behavioral aftereffect. These findings are consistent with the hypothesis that our subjective experience of time is represented by population coding in the right SMG. Future research is required to directly test the causal relationship between perceived duration and duration selectivity in the right SMG.
